# Anticalcification effects of DS-1211 in pseudoxanthoma elasticum mouse models and the role of tissue-nonspecific alkaline phosphatase in ABCC6-deficient ectopic calcification

**DOI:** 10.1038/s41598-022-23892-5

**Published:** 2022-11-18

**Authors:** Kaori Soma, Kengo Watanabe, Masanori Izumi

**Affiliations:** Daiichi Sankyo Co., Ltd, 1-2-58, Hiromachi, Shinagawa-Ku, Tokyo, 140-8710 Japan

**Keywords:** Diseases, Drug development, Preclinical research

## Abstract

Pseudoxanthoma elasticum (PXE) is a multisystem, genetic, ectopic mineralization disorder with no effective treatment. Inhibition of tissue-nonspecific alkaline phosphatase (TNAP) may prevent ectopic soft tissue calcification by increasing endogenous pyrophosphate (PPi). This study evaluated the anticalcification effects of DS-1211, an orally administered, potent, and highly selective small molecule TNAP inhibitor, in mouse models of PXE. Calcium content in vibrissae was measured in KK/HlJ and *ABCC6*^*-/-*^ mice after DS-1211 administration for 13–14 weeks. Pharmacokinetic and pharmacodynamic effects of DS-1211 were evaluated, including plasma alkaline phosphatase (ALP) activity and biomarker changes in PPi and pyridoxal-phosphate (PLP). Anticalcification effects of DS-1211 through TNAP inhibition were further evaluated in *ABCC6*^*-/-*^ mice with genetically reduced TNAP activity, *ABCC6*^*-/-*^/TNAP^+/+^ and *ABCC6*^*-/-*^/TNAP^+/-^. In KK/HlJ and *ABCC6*^*-/-*^ mouse models, DS-1211 inhibited plasma ALP activity in a dose-dependent manner and prevented progression of ectopic calcification compared with vehicle-treated mice. Plasma PPi and PLP increased dose-dependently with DS-1211 in *ABCC6*^*-/-*^ mice. Mice with *ABCC6*^*-/-*^/TNAP^+/-^ phenotype had significantly less calcification and higher plasma PPi and PLP than *ABCC6*^*-/-*^/TNAP^+/+^ mice. TNAP plays an active role in pathomechanistic pathways of dysregulated calcification, demonstrated by reduced ectopic calcification in mice with lower TNAP activity. DS-1211 may be a potential therapeutic drug for PXE.

## Introduction

Ectopic calcification is a pathological finding associated with both common disorders, such as diabetes and chronic kidney disease, and rare genetic diseases, like pseudoxanthoma elasticum (PXE) and generalized arterial calcification of infancy (GACI)^1,2^. PXE is an autosomal recessive disorder characterized by calcification of elastic fibers in skin, eyes, and blood vessels^3,4^. The pathomechanisms of PXE have been studied extensively; however, there is no established treatment for PXE^5^.

In many cases, PXE is caused by mutations in the adenosine triphosphate (ATP)-binding cassette subfamily C (*ABCC6*) gene^6^. ABCC6-dependent release of ATP from the liver is the main source of the potent mineralization inhibitor pyrophosphate (PPi) in plasma^4^. Patients with PXE demonstrate considerably reduced PPi levels, and this same trend has been shown in *ABCC6*^*-/-*^ mouse models^2,6^. Therefore, PPi deficiency has been identified as the underlying cause for PXE due to *ABCC6* mutations^2,6^. Therapeutics that increase plasma PPi levels are potential treatment candidates for PXE and other ectopic calcification disorders, as have been indicated in preclinical studies by Pomozi et al. and Dedinszki et al.^7,8^ Notably, some therapeutic medicines are currently under clinical development, such as recombinant ENPP1-Fc and oral PPi. This provides evidence of PPi as a promising target of anticalcification by multiple sources.

Tissue-nonspecific alkaline phosphatase (TNAP), a member of the alkaline phosphatase (ALP) family, is a key enzyme that regulates the homeostasis between physiological mineralization in skeletal and dental tissues and pathological ectopic calcification in soft tissues through hydrolysis of extracellular PPi^9,10^. Modulation of ALP enzymes, such as by TNAP inhibition, is an approach proposed to prevent ectopic soft tissue calcification by increasing PPi levels^2,11,12^. PPi is an endogenous substrate of TNAP, in addition to pyridoxal-phosphate (PLP; active form of vitamin B6) and phosphoethanolamine^13,14^. A number of TNAP inhibitors are described in the literature, including earlier non-specific inhibitors (ie, levamisole, theophylline) which show weak inhibition; and SBI-425, a potent and selective TNAP inhibitor, which shows robust in vivo activity but is not fully optimized for human clinical testing^10,15–17^. The specific role of TNAP in progression of ectopic mineralization in PXE is not well understood.

The *ABCC6*^*-/-*^ and KK/HlJ mouse models of PXE provide a system to explore investigational PXE treatment modalities. In *ABCC6*^*-/-*^ mice, ectopic connective tissue mineralization develops due to targeted ablation of the *ABCC6* gene, whereas KK/HlJ mice develop mineralization due to mutations in *ABCC6*^18–20^. These mouse models both provide similar ectopic calcification but with differing degrees of mineralization and associated phenotypes^18^. Studies utilizing these mouse models provide complementary insights into pathomechanisms of and potential treatments for PXE.

The role of TNAP in *ABCC6* deficiency and its contribution to calcification has been explored previously. Li et al. conducted experiments on *ABCC6*^*-/-*^ mice with heterozygous *ALPL*, the gene that encodes TNAP, to determine the effect of reduced TNAP activity on ectopic mineralization^2^. Results from this study showed *ABCC6*^*-/-*^/*ALPL*^+*/-*^ mice exhibited reduced plasma TNAP activity and reduced mineralization compared with *ABCC6*^*-/-*^/*ALPL*^+*/*+^; however, an increase in PPi due to *ABCC6*^*-/-*^/*ALPL*^+*/-*^ was not observed^2^.

DS-1211 is an orally administered, potent, and highly specific small molecule TNAP inhibitor. Preclinical studies described the pharmacological properties of DS-1211 and demonstrated it plays a role as a TNAP inhibitor in vivo; its potential to inhibit TNAP activity and increase plasma PPi showed that DS-1211 is suitable for administration to humans^21^. Two first-in-human, phase 1 studies on single and multiple ascending doses of DS-1211 found it is tolerated by healthy subjects over a wide range of doses^22^. The objectives of this study were to evaluate the anticalcification effects of the TNAP inhibitor DS-1211 in KK/HlJ and *ABCC6*^*-/-*^ mice and to confirm the contribution of TNAP inhibition to ectopic calcification through phenotype analysis of TNAP wild type (TNAP^+/+^) and TNAP heterozygous (TNAP^+/-^) *ABCC6*^*-/-*^ mice.

## Methods

### Animals

Five-week-old, male KK/HIJ and *ABCC6*^*-/-*^ mice were utilized in this study to investigate anticalcification effects of DS-1211. KK/HlJ mice were originally developed as part of a large-scale aging study that discovered these mice frequently manifest widespread tissue mineralization, particularly of the vibrissa wall, indicative of *ABCC6*^*-/-*^ mutations observed in PXE^23^. *ABCC6*^*-/-*^ mice were created by targeted ablation of the *ABCC6* gene and also demonstrate PXE-like ectopic mineralization^20^. Five-week-old, male C57BL/6J mice were also included in this study as the experimental control.

In the phenotype analysis experiment, six-week-old, male *ABCC6*^*-/-*^/TNAP normal, wild type mice (*ABCC6*^*-/-*^/TNAP^+/+^) and *ABCC6*^*-/-*^/TNAP heterozygous mice (*ABCC6*^*-/-*^/TNAP^+/-^) were studied. Eight-week-old, male C57BL/6J mice were also included as experimental controls.

All mice were purchased from Jackson Laboratory and expanded in Charles River Laboratories International Inc.; mice were acclimatized for 2–7 days. Experimental procedures were performed in Japan (Daiichi Sankyo Co., Ltd., Tokyo, Japan) and conducted in accordance with relevant guidelines and regulations approved by the appropriate ethics committee consisting of internal and independent external members (Institutional Animal Care and Use Committee, Daiichi Sankyo Co., Ltd.). All animal studies are reported in compliance with the ARRIVE 2.0 guidelines for reporting experiments involving animals^24^.

### Experimental design and DS-1211 administration

Table 1 describes the experimental groups for the animal models studied. Both the KK/HlJ and *ABCC6*^*-/-*^ experiments included a predose control group to demonstrate the state of ectopic calcification in the mice before compound administration. In the KK/HlJ mouse model experiment, DS-1211 was administered to mice by food admixture (ad libitum via lab chow). The compound was weighed and mixed with powder chow (FR-2, Funabashi Farm Co., Ltd) at concentrations of 0.0003% and 0.001% w/w. Mice were assigned to receive DS-1211 0.0003%, DS-1211 0.001%, or vehicle (powder chow only) for a 14-week period (99 days).

**Table 1:** Treatment groups in the mouse model experiments.

Experiment	Species	Treatment	Sample size, n	Experimental time point	Age at necropsy
Anticalcification in KK/HlJ mice	KK/HlJ	Predose	5	Week 0	6 weeks
	C57BL/6J	Vehicle	5	Week 14	20 weeks
	KK/HlJ	Vehicle	10	Week 14	20 weeks
	KK/HlJ	DS-1211 0.0003%	10	Week 14	20 weeks
	KK/HlJ	DS-1211 0.001%	10	Week 14	20 weeks
Anticalcification in *ABCC6*^*-/-*^ mice	C57BL/6J	Predose	5	Week 0	6 weeks
	*ABCC6*^*-/-*^	Predose	5	Week 0	6 weeks
	C57BL/6J	Vehicle	5	Week 13	19 weeks
	*ABCC6*^*-/-*^	Vehicle	10	Week 13	19 weeks
	*ABCC6*^*-/-*^	DS-1211 3 mg/kg	10	Week 13	19 weeks
	*ABCC6*^*-/-*^	DS-1211 10 mg/kg	10	Week 13	19 weeks
Analysis of TNAP^+/+^ and TNAP^+/-^ *ABCC6*^*-/-*^ mice	C57BL/6J	Pre-acceleration diet	8	Week 0	8 weeks
	*ABCC6*^*-/-*^/TNAP^+/+^	Pre-acceleration diet	10	Week 0	7 weeks
	*ABCC6*^*-/-*^/TNAP^+/-^	Pre-acceleration diet	10	Week 0	7 weeks
	C57BL/6J	Postacceleration diet	8	Week 14	22 weeks
	*ABCC6*^*-/-*^/TNAP^+/+^	Postacceleration diet	10	Week 14	21 weeks
	*ABCC6*^*-/-*^/TNAP^+/-^	Postacceleration diet	10	Week 14	21 weeks
	*ABCC6*^*-/-*^/TNAP^+/+^	Postacceleration diet	7	Week 33	40 weeks
	*ABCC6*^*-/-*^/TNAP^+/-^	Postacceleration diet	5	Week 33	40 weeks

*TNAP* tissue-nonspecific alkaline phosphatase.

In the *ABCC6*^*-/-*^ mouse model experiments, DS-1211 was weighed and dissolved in 0.5% w/v methyl cellulose solution (0.5% MC) at concentrations of 0.3 mg/mL and 1 mg/mL. Mice were randomly assigned to receive DS-1211 doses of 3 mg/kg, 10 mg/kg, or vehicle (0.5% MC) by oral gavage once daily for a 13-week period (91 days). The volume of administration was adjusted at 10 mL/kg based on the body weight. After randomization, *ABCC6*^*-/-*^ mice were provided with an acceleration diet (TD00442, Harlan Teklad) to promote calcification.

The experimental design and treatment groups for the phenotype analysis are also shown in Table 1. The *ABCC6*^*-/-*^/TNAP^+/+^ mice, *ABCC6*^*-/-*^/TNAP^+/-^ mice, and control mice in this experiment received an acceleration diet for 14 or 33 weeks to promote calcification (TD00442, Harlan Teklad).

### Calcium quantification

Calcium deposition was measured in vibrissae of KK/HlJ and *ABCC6*^*-/-*^ mice. In the DS-1211 anticalcification experiments, postdosing vibrissae were collected from KK/HlJ mice on Week 14 and from *ABCC6*^*-/-*^ mice on Week 13 after DS-1211 administration; predosing samples from KK/HlJ mice and from *ABCC6*^*-/-*^ mice were collected on Week 0^25^. After vibrissae were dried, they were homogenized in 10% formic acid, and calcium concentration in the homogenates was measured (Calcium Assay Kit, #Z5030014, BioChain). Calcium content (µg/mg dry weight of tissue) was calculated as (calcium concentration in the homogenates [mg/mL] x formic acid volume [mL])/(dry weight of tissue [mg] × 1000).

In the phenotype experiment, calcium deposition was measured in vibrissae of mice before and after the 14-week acceleration diet as described above, and in the aorta of two additional treatment groups after 33 weeks of the acceleration diet. The aorta samples were collected in *ABCC6*^*-/-*^/TNAP^+/+^ and *ABCC6*^*-/-*^/TNAP^+/-^ groups. Samples were dried and homogenized in 10% formic acid; calcium concentration in the homogenates was measured (Calcium Assay Kit, #Z5030014, BioChain).

### Plasma ALP activity assay

The plasma ALP activity assay used in this study was engineered to measure the degree of TNAP inhibition more accurately by minimizing plasma dilution in the total assay volume. In this study, plasma volume was 75% of the total assay volume, while in the standard clinical ALP assay, plasma volume is typically approximately 2% of the total assay volume.

Blood samples for the measurement of plasma ALP activity were collected from the tail veins of mice. In the DS-1211 anticalcification experiments, postdosing samples were collected from KK/HlJ mice on Week 14 and from *ABCC6*^*-/-*^ mice on Week 13, 30 min after the final dose of DS-1211; predosing samples were collected from KK/HlJ mice and from *ABCC6*^*-/-*^ mice on Week 0. In the phenotype analysis experiment, samples were collected from mice pre- and postacceleration diet at Week 0 and Week 14, respectively. Plasma samples were obtained through centrifugation and mixed with an assay buffer (50 mM Tris [pH 7.5], 1 mM MgCl_2_, 0.02 mM ZnCl_2_) and p-nitrophenylphosphate (1.55 mM) as the substrate. Samples were incubated at room temperature for 3 h. Absorbance pre- and postincubation were measured at 405 nm using a plate reader. Plasma ALP activity was calculated as the difference between pre- and postincubation absorbance.

### Quantification of plasma PPi and PLP concentrations

Blood samples were collected from the abdominal veins of mice for measurement of plasma PPi and plasma PLP concentrations. The mice were anesthetized, and blood for the measurement of plasma PPi and plasma PLP was collected carefully from the abdominal vein using an EDTA-treated syringe following euthanasia. The collected blood was put on ice soon after collection and samples were centrifuged within 10 min after blood collection. The collected blood was centrifuged at 9100 × g at 4 °C to separate the plasma. Plasma for the measurement of plasma PPi was further transferred to centrifugal filters (Merck-Millipore Ltd.) and then centrifuged under the same conditions to remove platelets. In the DS-1211 anticalcification experiments, postdosing samples were collected from KK/HlJ mice on Week 14 and from *ABCC6*^*-/-*^ mice on Week 13, 30 min after the final dose of DS-1211; predosing samples were collected from KK/HlJ mice and from *ABCC6*^*-/-*^ mice on Week 0. In the phenotype analysis experiment, samples were collected from mice pre- and postacceleration diet at Week 0 and Week 14, respectively.

For plasma PPi quantification, plasma samples were mixed with an assay mixture prepared with 2 mM MgCl_2_, 1 M HEPES buffer, 684 μg/mL adenosine 5’-phosphosulfate sodium salt, distilled water, and either adenosine triphosphate (ATP) sulfurylase (+ ATP_sul) or no ATP sulfurylase (− ATP_sul). Samples were mixed, centrifuged, and then incubated for 30 min at 37 °C, followed by 10 min at 90 °C. Next, samples were mixed and centrifuged at 3000 rpm for 20 min at 4 °C and added to a 96-well plate with 5 × CellTiter-Glo reagent. Luminescence was measured after a 10-min incubation at room temperature; samples were performed in duplicate. For plasma PLP quantification, plasma samples were treated using a protein precipitation method and analyzed using an internally validated liquid chromatography tandem mass spectrometry (LC/MS/MS) method. Plasma samples were mixed and heated at 40 °C for 30 min, followed by centrifugation at 20,000 g and 4 °C for 5 min. Sample supernatant was transferred to a 96-well plate and analyzed in an autosampler as an LC/MS/MS injection sample. The reproducibility and accuracy of these methods were confirmed internally prior to initiation of this study.

### Qualitative calcification analyses

Microcomputed tomography (microCT) imaging and histological analysis of vibrissae were conducted to visualize and qualitatively assess ectopic calcification. In the DS-1211 anticalcification experiments, microCT imaging was conducted at Week 13 in the C57BL/6 control, *ABCC6*^*-/-*^ control, and DS-1211 administered groups. In the phenotype analysis experiment, microCT imaging was conducted postacceleration diet (Week 14) in the control, *ABCC6*^*-/-*^/TNAP^+/+^, and *ABCC6*^*-/-*^/TNAP^+/-^ treatment groups. Mice were anesthetized and three-dimensional images of the calcified tissues around vibrissae were obtained (Rm_CT2-FX, Rigaku corporation, Tokyo, Japan) using the imaging parameters of 90 kV, 160 µA.

For the histological analysis, two mice from each treatment group were sacrificed before and after the 14-week acceleration diet. The vibrissae were fixed in 10% phosphate-buffered formalin, embedded in paraffin, and sectioned to a thickness of 4 µm. The Von Kossa method was used to stain slides and identify mineralization in the vibrissae^26^.

### Statistical analysis

Summary statistics including sample size (n), arithmetic mean (mean), and standard error (SE) were calculated for body weight, calcium content, plasma ALP activity, plasma DS-1211 concentration, and PLP concentrations (Microsoft Excel 2010, Microsoft Corporation). The median of plasma PPi concentration was calculated for each group.

In the DS-1211 anticalcification experiments, Dunnett’s test compared the body weight, calcium content, plasma ALP activity, and plasma PLP concentration of DS-1211 administered groups with the control group. Wilcoxon test compared plasma PPi concentration between control groups and predose treatment groups. Steel test compared the plasma PPi concentration of DS-1211 administered groups with the control group. Unpaired Student’s t-tests compared calcium content between control groups and predose treatment groups. A hypothesis test was performed using Spearmen’s rank correlation coefficient to test for the dose dependency of plasma ALP activity, plasma PPi concentration, plasma PLP concentration, and calcium content. In the phenotype experiment, unpaired Student’s t-tests compared calcium content, plasma ALP activity, and plasma PLP concentrations between experimental groups. Wilcoxon test compared plasma PPi concentrations between experimental groups. All statistical tests were performed in SAS^®^ (System Release 9.2, SAS Institute) and two-sided *P*-values < 0.05 were considered statistically significant.

## Results

### Anticalcification effects of DS-1211 in KK/HlJ mice

Administration of DS-1211 did not significantly affect the body weight of KK/HlJ mice during the experiment, and there was no significant difference in body weight between treatment groups. Table S1 shows the body weight of KK/HlJ mice at the end of the study. The body weights (mean ± SE) postdose on Week 14 in the DS-1211 0.0003% (40.6 ± 0.4 g) and DS-1211 0.001% (39.8 ± 0.8 g) groups were not significantly different from those of the vehicle-treated KK/HlJ control group (39.9 ± 0.7 g; *P* = 0.695 and *P* = 0.996, respectively).

**Figure 1:**
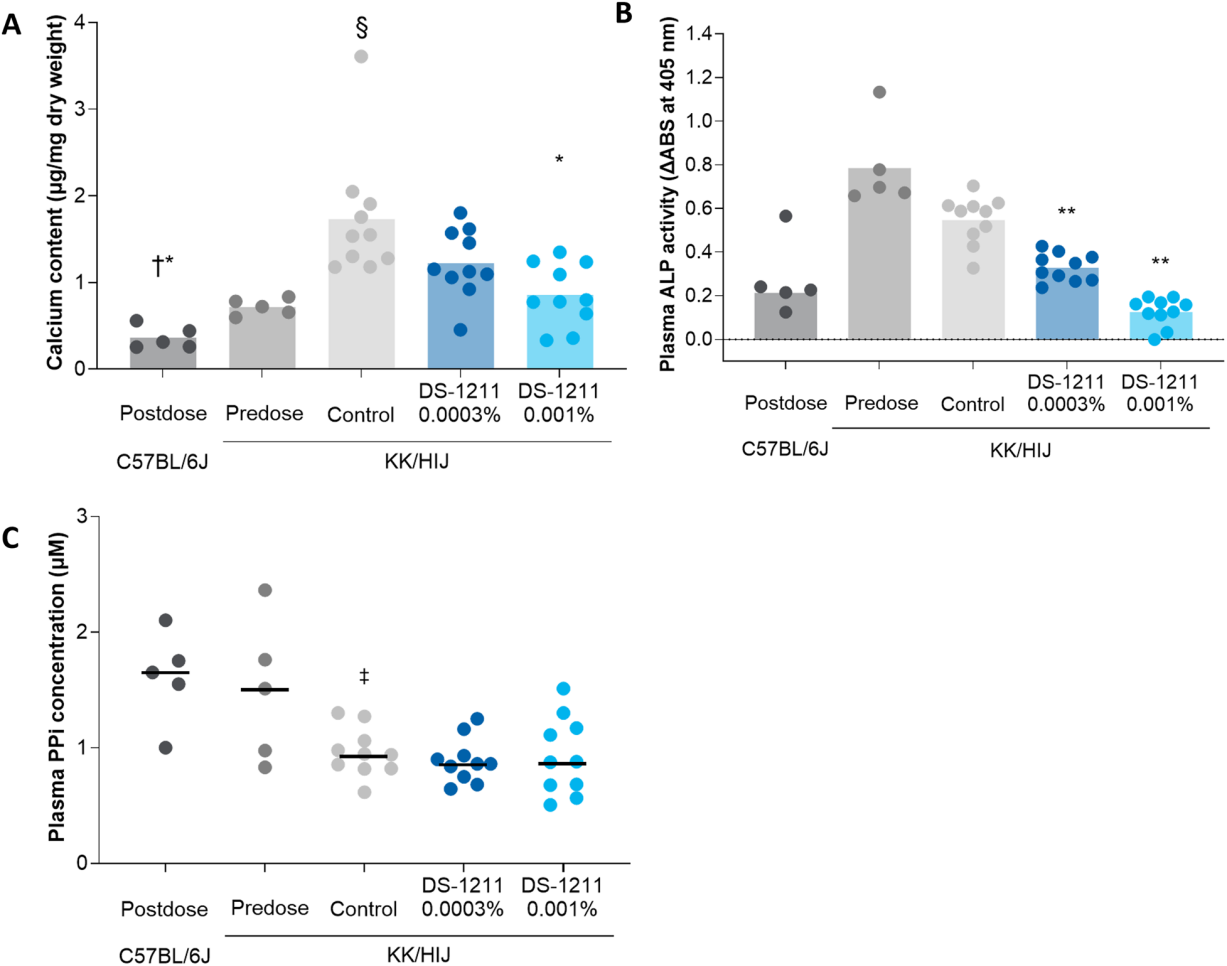
Calcium content (mean) (**A**), plasma ALP activity (mean) (**B**), and plasma PPi concentration (median) (**C**) in KK/HlJ and C57BL/6J mice before (KK/HlJ mice only) and after DS-1211 administration. *P < 0.005 versus control group; ^†^*P* < 0.005 versus pre-administration group; ^§^*P* < 0.01 versus pre-administration group; **P < 0.0001 versus control group; ^‡^*P* < 0.005 versus C57BL/6J group. ABS, absorbance; ALP, alkaline phosphatase; PPi, pyrophosphate.

Mean ± SE vibrissae calcium content was significantly higher in the postdose KK/HlJ control group (1.73 ± 0.23 µg/mg dry weight) compared with predose KK/HlJ mice (0.72 ± 0.04 µg/mg dry weight; *P* = 0.009; Fig. 1A). The postdose C57BL/6J group had significantly lower vibrissae calcium content (0.37 ± 0.06 µg/mg dry weight) than both the predose KK/HlJ mice (*P* = 0.001) and postdose KK/HlJ control mice (*P* = 0.001). DS-1211 0.001% significantly reduced vibrissae calcification progression in KK/HlJ mice. Vibrissae calcium content in the DS-1211 0.001% group postdose was 0.86 ± 0.12 µg/mg dry weight compared with 1.73 ± 0.23 µg/mg dry weight in the KK/HlJ control group (*P* = 0.002). There was no significant difference in vibrissae calcium content between the DS-1211 0.0003% group (1.23 ± 0.13 µg/mg dry weight) and the KK/HlJ control group (*P* = 0.069) after dosing.

Dose-dependent inhibition of plasma ALP activity was observed with the administration of DS-1211 (*P* < 0.0001). Both DS-1211 0.001% and DS-1211 0.0003% led to significant decreases in plasma ALP activity in KK/HlJ mice compared with the vehicle-treated KK/HlJ control group (*P* < 0.0001; Fig. 1B). Plasma ALP activity (mean ± SE) was inhibited 76.9% with DS-1211 0.001% compared with the KK/HlJ control (0.13 ± 0.02 vs. 0.55 ± 0.03); 39.9% plasma ALP inhibition was observed with DS-1211 0.0003% (0.33 ± 0.02).

Vehicle-treated KK/HlJ mice had significantly lower median plasma PPi concentration compared with the C57BL/6J control group (0.94 µM vs. 1.65 µM, respectively; *P* = 0.0047). There was no significant effect of DS-1211 on plasma PPi concentration in KK/HlJ mice (vehicle-treated KK/HlJ mice vs. DS-1211 0.0003% group, *P* = 0.5640; vehicle-treated KK/HlJ mice vs. DS-1211 0.001% group, *P* = 0.9354; Fig. 1C). Plasma PPi levels were numerically lower in the postdose vehicle-treated KK/HlJ mice compared with the predose KK/HlJ group, although this finding was not statistically significant (*P* = 0.0992).

### Anticalcification effects of DS-1211 in ABCC6^-/-^mice

Administration of DS-1211 did not affect the body weight of *ABCC6*^*-/-*^ mice during the experiment, and there was no significant difference in body weight between treatment groups. Table S1 shows the body weight of *ABCC6*^*-/-*^ mice at the end of the study. The postdose body weights (mean ± SE) on Week 13 in the DS-1211 3-mg/kg (30.3 ± 0.5 g) and DS-1211 10-mg/kg (30.4 ± 0.4 g) groups were not significantly different from those in the vehicle-treated *ABCC6*^*-/-*^ control group (30.6 ± 0.4 g; *P* = 0.906 and *P* = 0.979, respectively).

**Figure 2:**
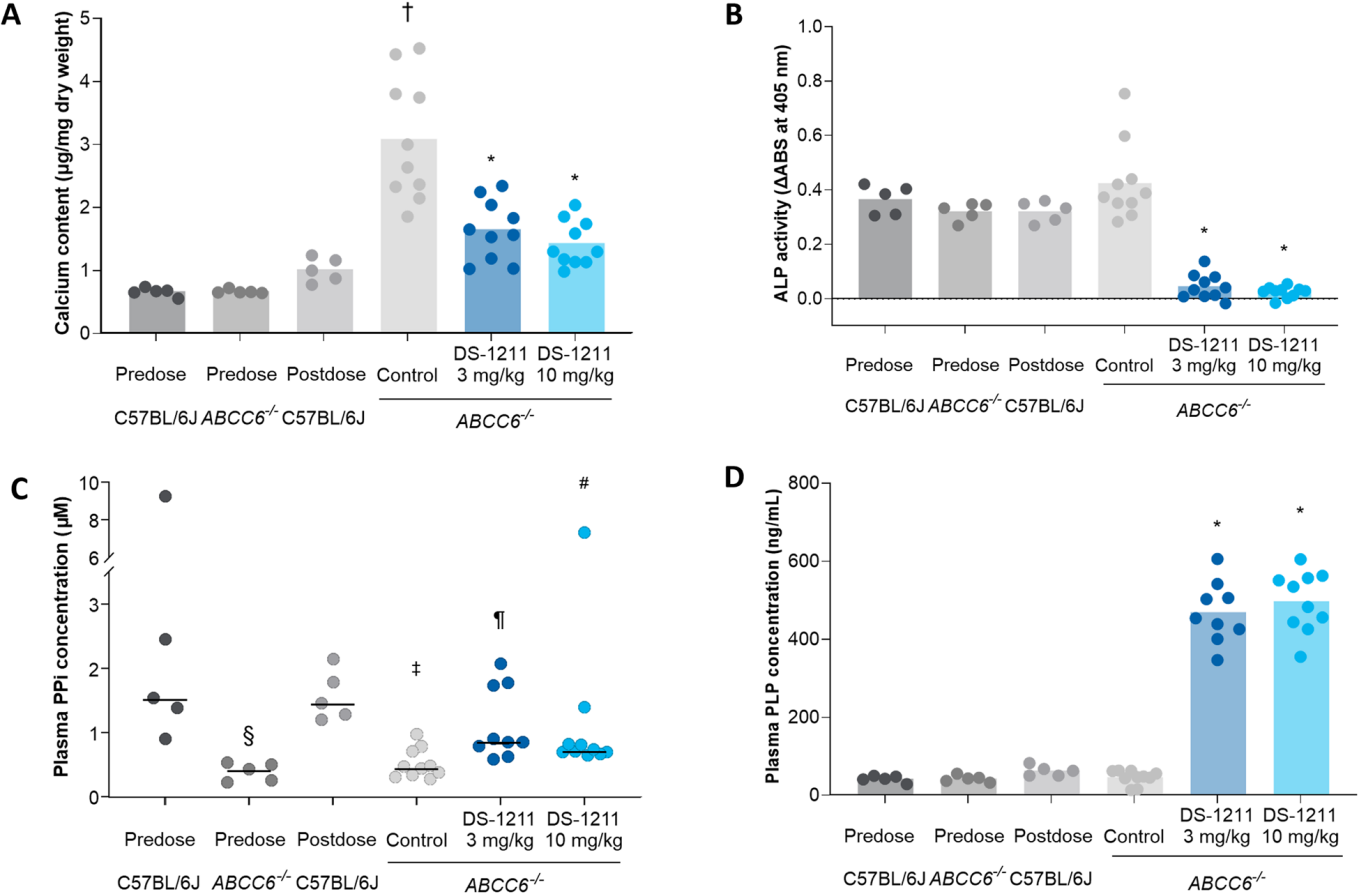
Calcium content (mean) (**A**), plasma ALP activity (mean) (**B**), plasma PPi concentration (median) (**C**), and plasma PLP concentration (mean) (**D**) in *ABCC6*^*-/-*^ and C57BL/6J mice before and after DS-1211 administration. *P < 0.0001 versus *ABCC6*^*-/*-^control group; ^†^*P* = 0.0005 versus postdose C57BL/6J control group; ^§^*P* < 0.01 versus C57BL/6J predose control group; ^‡^*P* < 0.001 versus C57BL/6J control group; ^¶^*P* < 0.01 versus *ABCC6*^*-/-*^ control group; ^#^*P* < 0.05 versus ABCC6^-/-^ control group. ABS, absorbance; ALP, alkaline phosphatase; PLP, pyridoxal-phosphate; PPi, pyrophosphate.

**Figure 3:**
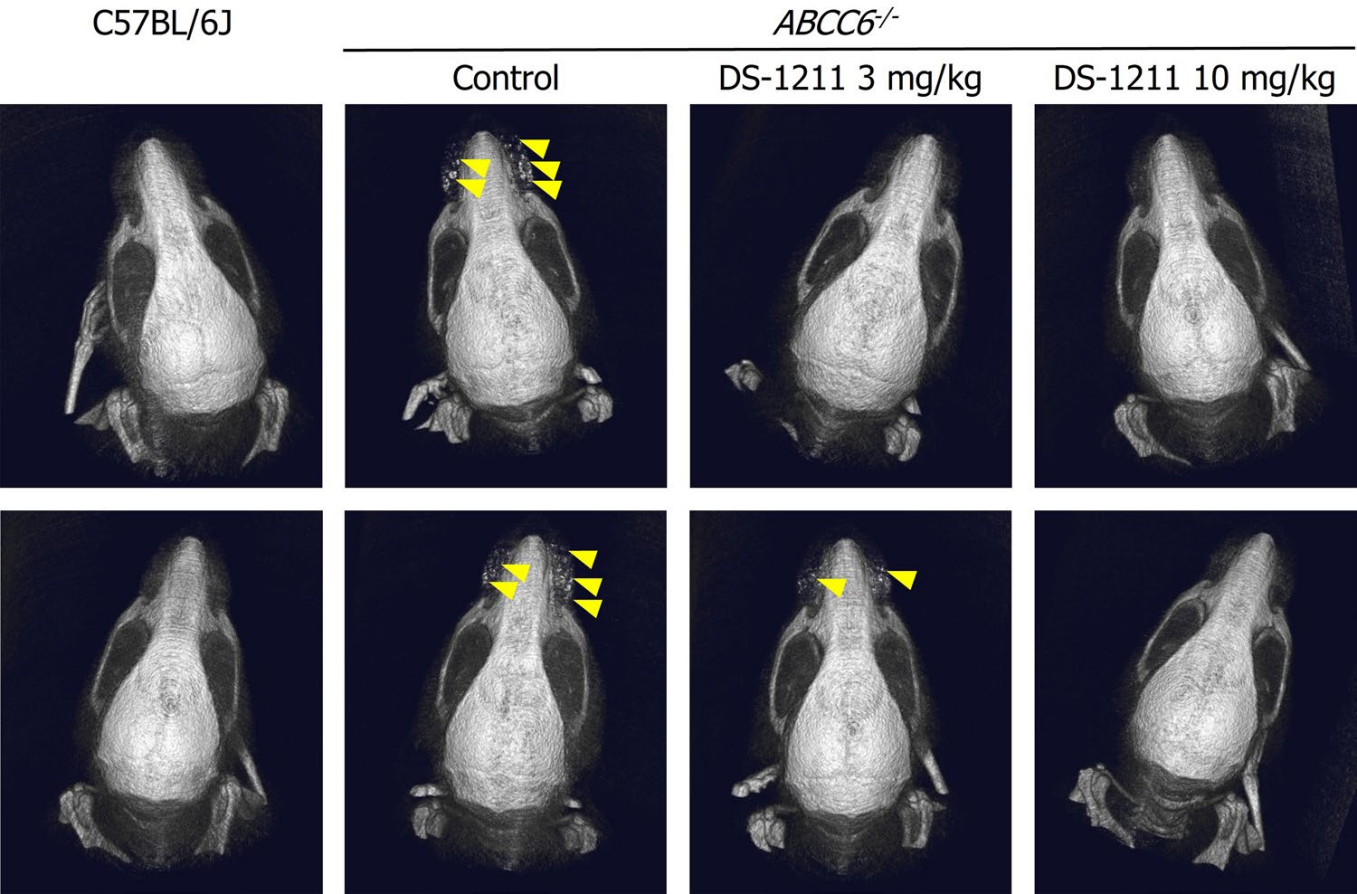
Microcomputed tomography imaging showing calcification of vibrissae in C57BL/6J and *ABCC6*^*-/-*^ control groups and *ABCC6*^*-/-*^ DS-1211 3 mg/kg and DS-1211 10 mg/kg treatment groups after dosing. Representative images from two mice in each group are shown. Yellow arrows indicate calcium deposition.

Vibrissae calcium content (mean ± SE) was significantly higher in the postdose *ABCC6*^*-/-*^ control group (3.08 ± 0.31 µg/mg dry weight) compared with predose *ABCC6*^*-/-*^ mice (0.67 ± 0.01 µg/mg dry weight; *P* = 0.0001; Fig. 2A). The postdose C57BL/6J group had significantly lower vibrissae calcium content (1.01 ± 0.09 µg/mg dry weight) than the postdose *ABCC6*^*-/-*^ control mice (*P* = 0.0005). *ABCC6*^*-/-*^ mice receiving DS-1211 3 mg/kg and DS-1211 10 mg/kg had dose-dependent reduction in vibrissae calcification progression compared with the vehicle-treated *ABCC6*^*-/-*^ control group (*P* < 0.0001). Calcium content measurements in the DS-1211 3-mg/kg and DS-1211 10-mg/kg groups after dosing were 1.65 ± 0.15 µg/mg dry weight and 1.43 ± 0.11 µg/mg dry weight, respectively (*P* < 0.0001 and *P* < 0.0001 vs. the *ABCC6*^*-/-*^ control group, respectively). Representative results from the microCT imaging of vibrissae postdosing are shown in Fig. 3. More calcium deposits were observed around vibrissae in the *ABCC6*^*-/-*^ control group compared with the C57BL/6J control group. Compared with the *ABCC6*^*-/-*^ control group, fewer calcium deposits were observed around vibrissae in the DS-1211 3 mg/kg and DS-1211 10 mg/kg groups.

Both DS-1211 3 mg/kg and DS-1211 10 mg/kg led to significant, dose-dependent decreases in plasma ALP activity compared with the *ABCC6*^*-/-*^ control group (*P* < 0.0001; Fig. 2B). Plasma ALP activity (mean ± SE) was inhibited 89.4% with DS-1211 3 mg/kg 30 min after oral dose administration compared with the *ABCC6*^*-/-*^ control (0.05 ± 0.01 vs. 0.42 ± 0.05, respectively; *P* < 0.0001); 94.2% plasma ALP inhibition was observed with DS-1211 10 mg/kg (0.02 ± 0.01).

DS-1211 led to numerical increases in plasma PPi (*P* = 0.226) and statistically significant dose-dependent increases in plasma PLP (*P* < 0.0001) concentrations 30 min after administration in *ABCC6*^*-/-*^ mice, as shown in Fig. 2C,D. In the predose *ABCC6*^*-/-*^ control group, median plasma PPi concentration (0.44 μM) was significantly lower than in the predose C57BL/6J group (1.54 μM, *P* = 0.008). After dosing, plasma PPi concentration remained lower in the *ABCC6*^*-/-*^ control group compared with the C57BL/6J group (*P* = 0.0007). Median plasma PPi concentration was significantly higher in both the DS-1211 3 mg/kg (0.86 μM) and DS-1211 10 mg/kg (0.73 μM) groups than in the postdose *ABCC6*^*-/-*^ control group (0.46 μM; *P* = 0.008 and *P* = 0.036, respectively; Fig. 2C). Plasma PLP concentration (mean ± SE) was significantly higher in both the DS-1211 3 mg/kg (469.3 ± 26.1 ng/mL) and DS-1211 10 mg/kg (497.5 ± 24.5 ng/mL) groups than in the postdose vehicle-treated *ABCC6*^*-/-*^ control group (45.8 ± 5.7 ng/mL; *P* < 0.0001 and *P* < 0.0001, respectively; Fig. 2D).

### Phenotype analysis of ABCC6^-/-^/TNAP^+/-^ mice

**Figure 4:**
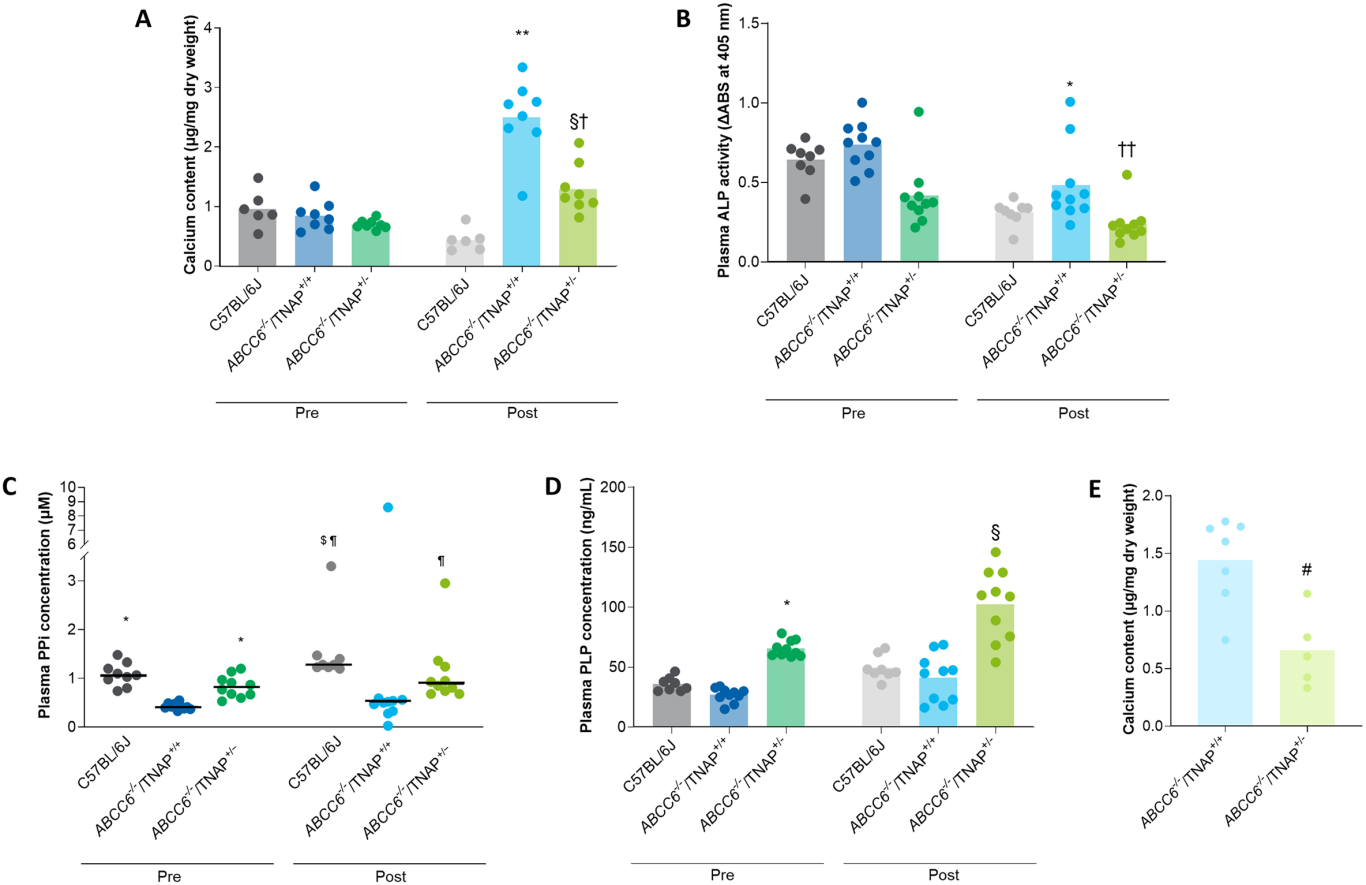
Calcium content (mean) of vibrissae (**A**), plasma ALP activity (mean) (**B**), plasma PPi concentration (median) (**C**), and plasma PLP concentration (mean) (**D**) in *ABCC6*^*-/-*^/TNAP^+/+^, *ABCC6*^*-/-*^/TNAP^+/-^, and C57BL/6J mice pre- and postacceleration diet; and calcium content (mean) of aorta in 40-week old *ABCC6*^*-/-*^/TNAP^+/+^ and *ABCC6*^*-/-*^/TNAP^+/-^ mice (**E**). *P < 0.001 versus pre-*ABCC6*^*-/-*^/TNAP^+/+^; ^†^*P* < 0.005 versus pre-*ABCC6*^*-/-*^/TNAP^+/-^; ^¶^*P* < 0.01 versus post-*ABCC6*^*-/-*^/TNAP^+/+^; ^§^*P* < 0.001 versus post-*ABCC6*^*-/-*^/TNAP^+/+^; ^$^*P* < 0.05 versus post-*ABCC6*^*-/-*^/TNAP^+/-^; ^#^*P* < 0.005 versus 40-week *ABCC6*^*-/-*^/TNAP^+/+^; **P < 0.0001 versus pre-*ABCC6*^*-/-*^/TNAP^+/+^; ^††^P < 0.05 versus pre-*ABCC6*^*-/-*^/TNAP^+/+^. ABS, absorbance; ALP, alkaline phosphatase; PLP, pyridoxal-phosphate; PPi, pyrophosphate; TNAP, tissue-nonspecific alkaline phosphatase.

**Figure 5:**
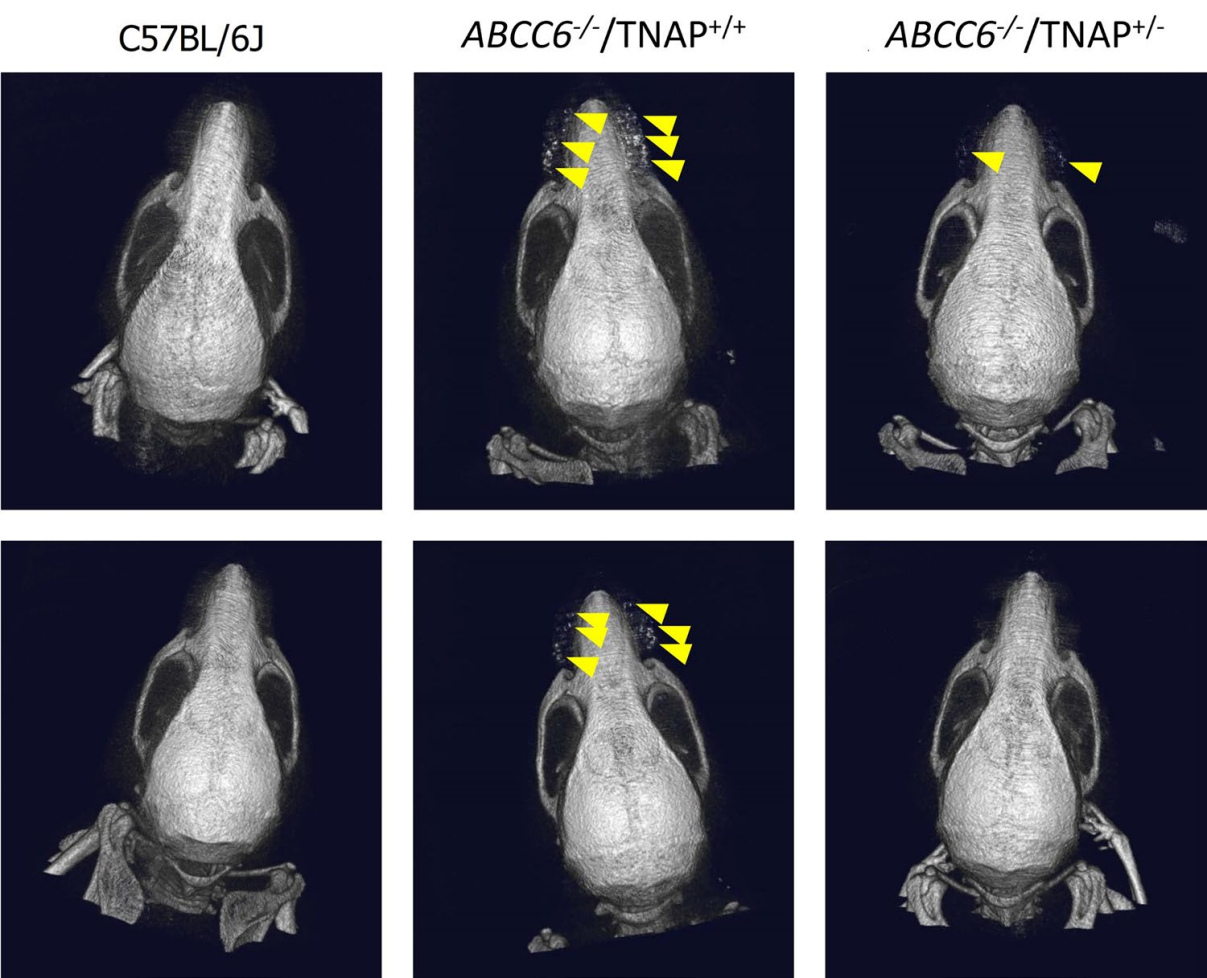
Microcomputed tomography imaging showing calcification of vibrissae in *ABCC6*^*-/-*^/TNAP^+/+^ mice, *ABCC6*^*-/-*^/TNAP^+/-^ mice, and control C57BL/6J mice after the acceleration diet. Representative images from two mice in each group are shown. Yellow arrows indicate calcium deposition. TNAP, tissue-nonspecific alkaline phosphatase.

**Figure 6:**
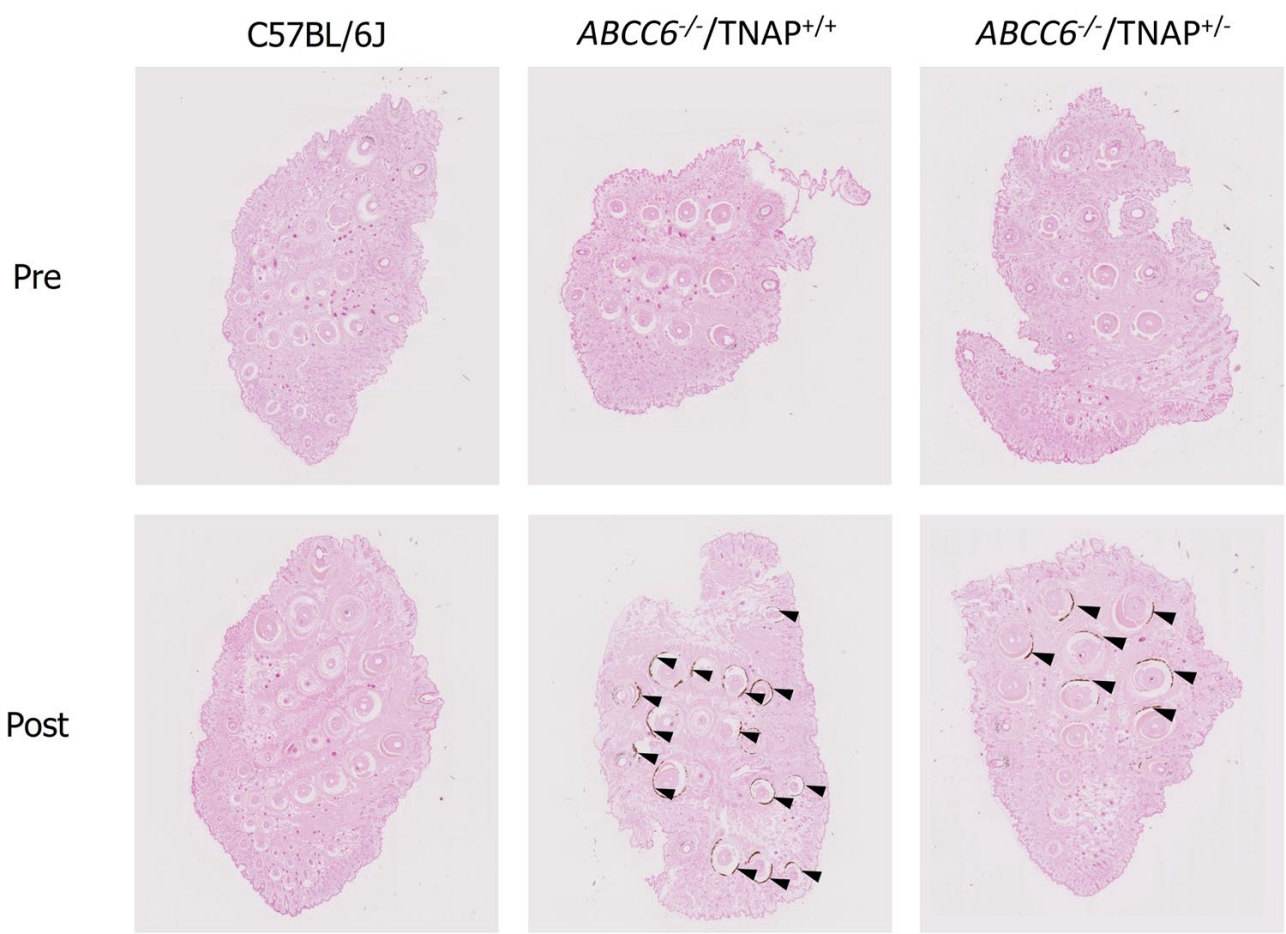
Histological analysis with Von Kossa staining showing calcification of vibrissae in *ABCC6*^*-/-*^/TNAP^+/+^ mice, *ABCC6*^*-/-*^/TNAP^+/-^ mice, and control C57BL/6J mice pre- and postacceleration diet. TNAP, tissue-nonspecific alkaline phosphatase.

After 14 weeks of being fed an acceleration diet, calcium content (mean ± SE) in *ABCC6*^*-/-*^/TNAP^+/+^ mice increased from 0.85 ± 0.09 dry weight to 2.50 ± 0.23 µg/mg dry weight (*P* < 0.0001); this increase was diminished in *ABCC6*^*-/-*^/TNAP^+/-^ mice (0.70 ± 0.03 to 1.30 ± 0.15 µg/mg dry weight; *P* = 0.0012). The TNAP^+/-^ phenotype reduced *ABCC6*-induced ectopic calcification. *ABCC6*^*-/-*^/TNAP^+/-^ mice had significantly less calcification postacceleration diet compared with normal *ABCC6*^*-/-*^/TNAP^+/+^ mice (*P* = 0.0005; Fig. 4A). Decreased calcification of vibrissae in *ABCC6*^*-/-*^/TNAP^+/-^ mice compared with *ABCC6*^*-/-*^/TNAP^+/+^ mice was observed through microCT imaging and histological analysis. After the acceleration diet, calcium deposits around the vibrissae were observed in *ABCC6*^*-/-*^/TNAP^+/+^ mice, whereas little to no calcium deposits were observed in the *ABCC6*^*-/-*^/TNAP^+/-^ and control groups (Figs. 5 and 6). In two additional groups, calcium content was measured in the aorta after a 33-week acceleration diet. Calcium in the aorta was also significantly lower in the 40-week old *ABCC6*^*-/-*^/TNAP^+/-^ group (0.66 ± 0.15 μg/mg dry weight) compared with the 40-week old *ABCC6*^*-/-*^/TNAP^+/+^ group (1.44 ± 0.14 µg/mg dry weight; *P* = 0.004; Fig. 4E).

Biomarker analysis showed the TNAP^+/-^ phenotype inhibited plasma ALP activity and increased plasma PPi concentration and plasma PLP concentration. Plasma ALP activity (mean ± SE) was significantly lower in *ABCC6*^*-/-*^/TNAP^+/-^ mice compared with *ABCC6*^*-/-*^/TNAP^+/+^ mice both pre- (0.42 ± 0.06 vs. 0.74 ± 0.0463, respectively; *P* = 0.0007) and postacceleration diet (0.24 ± 0.04 vs. 0.48 ± 0.08, respectively; *P* = 0.0114; Fig. 4B). Median plasma PPi concentration was significantly higher in *ABCC6*^*-/-*^/TNAP^+/-^ mice compared with *ABCC6*^*-/-*^/TNAP^+/+^ mice before (0.82 µM vs. 0.41 µM, respectively; *P* = 0.0004) and after the acceleration diet (0.89 µM vs. 0.52 µM, respectively; *P* = 0.0049; Fig. 4C). Additionally, *ABCC6*^*-/-*^/TNAP^+/-^ mice had significantly higher plasma PLP concentration than *ABCC6*^*-/-*^/TNAP^+/+^ mice at both experimental time points (Fig. 4D). Before the acceleration diet, mean ± SE plasma PLP concentration was 65.6 ± 2.2 ng/mL in the *ABCC6*^*-/-*^/TNAP^+/-^ group and 26.9 ± 1.9 ng/mL in the *ABCC6*^*-/-*^/TNAP^+/+^ group (*P* < 0.0001); the same trend was observed after the acceleration diet (102.3 ± 9.4 ng/mL and 41.0 ± 6.2 ng/mL, respectively; *P* < 0.0001).

## Discussion

Potential therapeutic strategies to prevent ectopic soft tissue calcification involve inhibition of TNAP to increase the level of the potent endogenous anticalcification factor PPi^9,12^. Here, we report the anticalcification effects of DS-1211, an orally administered, potent, and highly specific small molecule TNAP inhibitor, in KK/HlJ and *ABCC6*^*-/-*^ mouse models of PXE. Additionally, mice with phenotype *ABCC6*^*-/-*^/TNAP^+/+^ and *ABCC6*^*-/-*^/TNAP^+/-^ were studied to elucidate the feasibility of TNAP inhibition as a therapeutic target for ectopic calcification by means of increasing PPi levels. In KK/HlJ and *ABCC6*^*-/-*^ mouse models, administration of DS-1211 resulted in dose-dependent decreases in plasma ALP activity and prevented the progression of ectopic calcification compared with vehicle-treated mice. In the *ABCC6*^*-/-*^ mouse model, administration of DS-1211 also increased plasma PPi concentration and plasma PLP concentration, two substrates hydrolyzed by TNAP. The phenotype analysis in *ABCC6*^*-/-*^/TNAP wild type and *ABCC6*^-/-^/TNAP^+/-^ mice demonstrated that TNAP^+/-^ reduced *ABCC6*-induced ectopic calcification, inhibited plasma ALP activity, and increased the concentrations of plasma PPi and plasma PLP compared with *ABCC6*^*-/-*^/TNAP^+/+^ mice. These results suggest TNAP plays an active role in pathomechanistic pathways of ectopic calcification, and a TNAP inhibitor like DS-1211 may be a promising therapeutic drug for ectopic mineralization in PXE.

In KK/HlJ mice, a mouse model of spontaneous PXE, there was no effect of DS-1211 on plasma PPi levels despite significantly reduced calcium content and inhibited plasma ALP activity. In contrast, *ABCC6*^*-/-*^ mice demonstrated increased plasma PPi and plasma PLP levels with DS-1211 administration and simultaneously had reduced calcium content and plasma ALP activity. This difference in the effect of DS-1211 on plasma PPi levels between KK/HlJ and *ABCC6*^*-/-*^ mice may be related to a difference in the DS-1211 administration route between the two experiments. KK/HlJ mice were administered DS-1211 by food admixture, meaning that animals ate food freely and took the compound ad libitum. Conversely, *ABCC6*^*-/-*^ mice received DS-1211 by oral gavage. Compound administration by food admixture leads to more mild, gradual, and sustained compound exposure that may vary in degree among individuals depending on food intake; administration by oral gavage induces acute, rapid, and shorter exposure to the compound that is consistent in degree among individuals. The administration route may have also affected the rate of ALP inhibition between KK/HlJ and *ABCC6*^*-/-*^ mice. *ABCC6*^*-/-*^ mice had higher maximum ALP inhibition rates 30 min after administration (90%–95%) than the KK/HlJ mice (76.9%). Lower ALP inhibition in the KK/HlJ mice may have contributed to the lack of increased plasma PPi observed in this experiment. These findings suggest that plasma PPi concentrations may be affected by the method of DS-1211 administration and the subsequent compound exposure.

As plasma ALP levels are closely related to osteoblastic activity and bone growth, ALP activity is generally higher in younger individuals^27^. Findings in the current study align with this phenomena, as predose KK/HlJ mice (6 weeks of age at necropsy) had numerically higher plasma ALP activity compared with that of the postdose, vehicle-treated C57BL/6J mice and postdose, vehicle-treated control KK/HlJ mice (both 20 weeks of age at necropsy; Fig. 1B). Notably, we also observed a numerical difference in plasma ALP activity between the postdose, vehicle-treated C57BL/6J mice and postdose, vehicle-treated control KK/HlJ mice (both 20 weeks of age at necropsy). The cause of this difference is unknown; there may be a difference in basal serum ALP activity between the two strains.

Notably, DS-1211 inhibited the progression of ectopic calcification in both KK/HlJ and *ABCC6*^*-/-*^ mouse models, and this effect occurred in KK/HlJ mice that had no increase in plasma PPi concentration. This suggests that PPi in the vibrissae of the DS-1211 administered KK/HlJ group was increased based on the observed anticalcification effect. Anticalcification effects driven by PPi may depend on the concentration of PPi in local tissues rather than in plasma, although more research is needed to confirm this hypothesis. Li et al. examined the effect of TNAP inhibition on plasma PPi levels and ectopic calcification in *ABCC6*^*-/-*^ mice and did not find a strong correlation between ectopic calcification and plasma PPi levels, which may support this hypothesis^2^.

The phenotype analysis of *ABCC6*^*-/-*^/TNAP^+/+^ mice and *ABCC6*^*-/-*^/TNAP^+/-^ mice isolated the effect of TNAP on ectopic calcification due to *ABCC6* deficiency and removed the variability of differing mouse models and experimental methods. Our results agree with the literature; a study on *ABCC6*^*-/-*^ mice with heterozygous *ALPL*, the gene that encodes TNAP, researched the effect of reduced TNAP activity on ectopic mineralization^2^. They found *ABCC6*^*-/-*^/*ALPL*^+*/-*^mice exhibited reduced plasma TNAP activity and reduced mineralization compared with *ABCC6*^*-/-*^/*ALPL*^+*/*+^; however, an increase in PPi due to *ABCC6*^*-/-*^/*ALPL*^+*/-*^ was not observed^2^. In our study, a difference in plasma PPi was observed between *ABCC6*^*-/-*^/TNAP^+/+^ and *ABCC6*^*-/-*^/TNAP^+/-^ mice, although the degree of difference was much smaller than that observed in mice receiving DS-1211 by oral gavage. This may be due to differences in genetical background of mice strain used in each study, although as previously mentioned, an increase in PPi resulting from 50% ALP inhibition may be very difficult to detect, especially in the plasma.

Moreover, we built upon the previous work and further investigated ectopic calcification of the aorta in *ABCC6*^*-/-*^/TNAP^+/+^ and *ABCC6*^*-/-*^/TNAP^+/-^ mice. Ectopic calcification in the aorta caused by ABCC6 deficiency was observed in mice old in age. As investigations of the *ABCC6*^*-/-*^ mouse shows it develops a phenotype with mineralization similar to that of PXE patients, our findings may reflect that aortic calcification of PXE patients occurs after the appearance of other symptoms, such as those in the eyes and skin^20^. An early study investigating the phenotype of *ABCC6*^*-/-*^ mice noted that blood vessel calcification first appeared when mice were around 6 months old and progressed with age; the older mice exhibited calcification in the aortic tissues^28^. We observed ectopic calcification in the aorta of this PXE mouse model and found lower calcium content in the aorta of *ABCC6*^*-/-*^/TNAP^+/-^ mice than in *ABCC6*^*-/-*^/TNAP^+/+^ mice. Our results, combined with these previously published data, provide further support that the inhibition of TNAP is a promising therapeutic strategy to prevent ectopic mineralization in the whole body in PXE.

A limitation of these experiments is that calcification of the eyes and skin was not examined. Additionally, KK/HlJ and *ABCC6*^*-/-*^ mice were fed different diets; this decision was based on internal research, which found an acceleration diet was essential for ectopic calcification in *ABCC6*^*-/-*^ mice but was not needed for ectopic calcification in KK/HlJ mice. This result agrees with the literature that shows ectopic calcification progresses in KK/HlJ mice with aging without the necessity of acceleration diet^18,23^. Furthermore, interpretations of these results are limited by the preclinical scope of the studies and relatively low sample sizes in experimental groups. Larger group sample sizes may have better accounted for the substantial variation in PPi among individuals. Clinical studies are needed to establish the efficacy and safety of DS-1211 as a therapeutic strategy to prevent the progression of ectopic calcification in PXE patients. The risks of overly inhibited TNAP should be considered, as nearly complete or complete loss of TNAP activity is associated with the development of hypophosphatasia, a rare mineralization disorder often leading to rickets, osteomalacia, or hypomineralization of teeth^29^. Lastly, although this study did not examine the effect of DS-1211 on bone, previously conducted toxicity studies in mice, rats, and monkeys demonstrated that no significant effect was present, especially at the pharmaceutically active dosage.

DS-1211, an orally administered, potent, and highly specific TNAP inhibitor prevented the progression of ectopic calcification in KK/HlJ and *ABCC6*^*-/-*^ mouse models of PXE. These results suggest DS-1211 may be a potential treatment for PXE. Further investigation of the efficacy and safety of DS-1211 in clinical studies with PXE patients are warranted.

## Data availability

All data relevant to the study are included in the article and Supplementary Material. Additional data may be available from the corresponding author upon request. Some additional data may not be made available because of proprietary restrictions.

## Supplementary Information

Supplementary Information.
