# Supplementary material for: Anticalcification effects of DS-1211 in pseudoxanthoma elasticum mouse models and the role of tissue-nonspecific alkaline phosphatase in ABCC6-deficient ectopic calcification
Source: Sci Rep. 2022 Nov 18;12:19852. doi: 10.1038/s41598-022-23892-5 (PMC9674622; doi:10.1038/s41598-022-23892-5)
Supplement: Supplementary file 1 — Supplementary Information. [file 41598_2022_23892_MOESM1_ESM.pdf]

Anticalcification effects of DS-1211 in pseudoxanthoma elasticum mouse models and the role of tissue-nonspecific alkaline phosphatase in ABCC6-deficient ectopic calcification

Kaori Soma,*[1] Kengo Watanabe,[1] Masanori Izumi[1]

[1]Daiichi Sankyo Co., Ltd., Tokyo, Japan

Supplementary Material

Table S1. Body weight of KK/HlJ mice and *ABCC6*$^{-/-}$ mice after DS-1211 administration

	**Body weight (g), mean ± SE**	***P*-value vs control**
**KK/HlJ mice**		
**KK/HlJ control**	39.9 ± 0.7	
**DS-1211 0.0003%**	40.6 ± 0.4	0.695
**DS-1211 0.001%**	39.8 ± 0.8	0.996
***ABCC6*$^{-/-}$ mice**		
***ABCC6*$^{-/-}$ control**	30.6 ± 0.4	
**DS-1211 3 mg/kg**	30.3 ± 0.5	0.906
**DS-1211 10 mg/kg**	30.4 ± 0.4	0.979

SE, standard error.
